# Attitudes and experiences towards the application of motivational interviewing by podiatrists working with people with diabetes at high-risk of developing foot ulcers: a mixed-methods study

**DOI:** 10.1186/s13047-022-00567-y

**Published:** 2022-08-19

**Authors:** M. Jongebloed-Westra, C. Bode, B. E. Bente, J. M. de Jonge, P. M. ten Klooster, H. Koffijberg, S. H. Exterkate, J. J. van Netten, J. E. W. C. van Gemert-Pijnen

**Affiliations:** 1 Department of Psychology, Health and Technology, Centre for eHealth Research and Wellbeing, University of Twente, PO Box 217, 7500 AE Enschede, The Netherlands; 2 ACHIEVE, Centre of Applied Research, Faculty of Health, Amsterdam University of Applied Sciences, Amsterdam, The Netherlands; 3 Department of Health Technology and Services Research, Tech Med Centre, University of Twente, PO Box 217, 7500 AE Enschede, The Netherlands; 4 Voetencentrum Wender, Sabina Klinkhamerweg 10, 7555 SK Hengelo, The Netherlands; 5 Voetmax Orthopedie, Sabina Klinkhamerweg 10, 7555 SK Hengelo, The Netherlands; 6 Department of Rehabilitation, Amsterdam UMC, Location Academic Medical Centre, University of Amsterdam, Amsterdam Movement Sciences, Meibergdreef 9, 1105 AZ Amsterdam, The Netherlands; 7 Diabetic Foot Unit, Department of Surgery, Hospital Group Twente, PO Box 7600, 7600 SZ Almelo, The Netherlands

**Keywords:** Diabetes mellitus, Diabetic foot, Motivational interviewing, Communication, Training, Podiatry, Behavioural change, Adherence

## Abstract

**Background:**

Podiatrists are key professionals in promoting adequate foot self-care for people with diabetes at high-risk of developing foot ulcers. However, merely informing patients about the advantages of foot self-care is insufficient to realise behavioural change. Motivational interviewing (MI) is a promising person-centred communication style that could help to create a working alliance between healthcare providers and patient to improve foot self-care. This study aims to observe and analyse the application of MI in consultations carried out by MI-trained and non-MI-trained podiatrists with their patients, and explore podiatrists’ attitudes and experiences towards MI.

**Methods:**

Eighteen podiatrists (median age: 28.5 years, 10 female and 8 male) followed a three-day basic training in MI and 4 podiatrists (median age: 38.5 years, 4 female) were not trained in MI. To observe and rate the MI-fidelity in daily clinical practice, audio recordings from the MI-trained and non-MI-trained podiatrists were scored with the Motivational Interviewing Treatment Integrity code. Individual, semi-structed, in-depth interviews were conducted with the MI-trained podiatrists to explore their attitudes towards and experiences with MI. These data sources were triangulated to describe the effect of training podiatrists in MI for their clinical practice.

**Results:**

The MI-trained podiatrists scored significantly higher than the non-MI-trained podiatrists on two of four global MI-related communication skills (empathy, *p* = 0.008 and change talk, *p* = 0.008), on one of five core MI-adherent behaviours (affirmation, *p* = 0.041) and on one of the other behaviour counts (simple reflections, *p* = 0.008). The podiatrists mainly reported their attitudes and experiences regarding partnership and cultivating change talk, during the interviews. In addition, they also mentioned facilitators and barriers to using MI and indicated whether they experienced MI as having added value.

**Conclusions:**

The MI-trained podiatrists used the principles of MI at a solid beginner proficiency level in their clinical practice in comparison to the non-MI-trained podiatrists, who did not reach this level. This achievement is in accordance with the basic MI-training they received. This multi-method study reveals that podiatrists can be effectively trained in applying MI in daily clinical practice.

**Trial registration:**

Netherlands Trial Register NL7710. Registered: 6 May 2019.

**Supplementary Information:**

The online version contains supplementary material available at 10.1186/s13047-022-00567-y.

## Background

People with diabetes have a 19–34% lifetime risk of developing foot ulcers [1]. Diabetes-related foot ulcers [2] can lead to foot infection, amputation, hospitalisation, immobility and a reduced quality of life [1, 3]. Therefore, people at high-risk of developing foot ulcers are recommended to see a podiatrist once every 1–3 months, as compared to every 12 months or less for those not at high-risk [4]. In addition to podiatric medical care, stimulation and facilitation of behavioural change and adherence to foot self-care are crucial to improve the’ outcomes for people at high-risk of developing foot ulcers [1, 5–8]. Behavioural change and adherence to foot self-care includes wearing orthopaedic shoes, attending foot-care appointments, not walking barefoot, and daily self-monitoring of foot temperature and for signs of impending ulceration [1, 5, 7]. Since podiatrists provide long-term foot care to people with diabetes, they are a key professional in for stimulating behavioural change and adherence to foot self-care [9–11].

Adherence is defined as the extent to which a person’s behaviour corresponds with agreed recommendations for treatment from a healthcare provider [12]. To improve foot self-care, patient education is often used to increase the person’s skills and knowledge [4, 8, 13, 14]. However, previous studies also show that merely informing at-risk people with diabetes about the advantages of foot self-care is insufficient to realise behavioural change [13–17]. While people at high risk of foot ulcer generally have the required knowledge about prevention and risks [13, 18], and a high perceived self-efficacy [13, 18], their adherence to self-care behaviour is consistently low [4, 5, 19, 20]. Therefore, techniques other than mere knowledge transfer are important to stimulate behavioural change and improve adherence to foot self-care.

One key factor to change behaviour is a person’s motivation. Montano et al. [21] described that recommended behaviours must be considered important enough by that person for them to become adherent to these behaviours. A healthcare provider’s communication style and behaviour can affect a person’s motivation to become adherent, and thus contribute to behavioural change [13, 22, 23]. To influence adherence to foot self-care behaviour, a working alliance or partnership between the podiatrist and patient is needed. A working alliance or partnership means that the podiatrist and patient have to work together to increase adherence by changing the person’s behaviour regarding foot self-care. This is instead of the podiatrist using traditional health education approaches such as taking the expert role, thereby that negatively impacting the change of behaviour [22, 23]. However, until now, most podiatrists still use a traditional communication style in patient education, which is directive and one-sided, and focused on giving expert advice [24, 25]. Like other healthcare providers, podiatrists generally receive communication training, but not specifically on building working alliances with their patients or specific person-centred communication techniques to elicit behavioural change and avoid resistant reactions in people [24, 26]. Without developing these shared decision-making skills, podiatrists have a reduced effectiveness for changing the foot self-care behaviour of people at high-risk of developing foot ulcers. Podiatrists could apply a more person-centred approach with shared decision-making, in which behavioural change is the aim [27–30], by learning to listen to and engage with patient perspectives of their own situation. Furthermore, podiatrists can discuss patient expectation and acceptance of the recommended treatment.

Motivational interviewing (MI) is one promising person-centred communication style designed to stimulate and enhance behavioural change. MI is a collaborative, goal-oriented style of communication with particular attention on the language of change. MI consists of two active components: a relational component, which focuses on empathy and the interpersonal spirit of MI, and a technical component, which involves the differential evocation and reinforcement of client change [31]. MI is designed to strengthen personal motivation and commitment to a specific goal by eliciting and exploring a person’s own reasons for change within an atmosphere of acceptance and compassion [32]. MI requires the healthcare provider to engage in a working alliance with the patient as equal partner, and use communication skills that stimulate behavioural changes. This is without giving unsolicited advice or directing, confronting, warning, or instructing the patient. Systematic reviews and meta-analyses show that MI has been used successfully in a wide array of health behaviour or lifestyle problems, and has demonstrated robust effects in a variety of clinical settings and diseases [33–38]. However, it has also been shown in various healthcare contexts that mastering MI requires training and practice [39, 40] and that time investment, self-awareness and discipline from the healthcare provider are needed to apply an MI-communication style [41]. This also applies to diabetes healthcare providers [42].

Recently Kaczmarek and colleagues found that training podiatrists in MI has the potential to improve their MI-related communication skills [25]. These podiatrists showed a small increase in MI-related skills two weeks after training, however, these changes were short-lived and 12 weeks after training improvements were no longer detectable [25]. In addition, another explorative study that used a short, feedback-driven training program showed that the investigators were sufficiently trained to enhance motivation for change in people with diabetes at high-risk of foot ulcers [41]. However, Kaczmarek et al. conducted their study without a control group [25] and Keukenkamp et al. trained investigators who had no direct clinical experience in MI instead of using podiatrists [43].

Therefore, our aims were to analyse the application of MI in consultations carried out by MI-trained and non-MI-trained podiatrists in daily clinical practice, and to explore the podiatrists’ attitudes and experiences towards the use of MI and the implementation of the MI-techniques in their work with people with diabetes at high-risk of foot ulcers.

## Methods

This study is part of a randomised controlled trial (RCT) examining the effectiveness of using MI combined with digital shoe-fitting to improve adherence to wearing orthopaedic shoes [44]. In the RCT, patients were randomised over the intervention (motivational interviewing + digital shoe-fitting) and control (usual care) condition at the level of the treating podiatrist. All patients in the intervention group received one face-to-face MI-appointment with their MI-trained podiatrist in addition to their usual appointments. The study was exempt from full medical ethical approval by the CMO region Arnhem – Nijmegen (NL 68,567.091.19), because the CMO judged that the participants in the RCT were imposed to such actions or behaviours that the study was not regarded to fall under the Medical Research Involving Human Subjects Act (WMO). Besides this, in accordance with the Medical Device Regulation (MDR) the study did not require a positive recommendation by the CMO, because the sensor used in the study has a CE marking, and the sensor and software were not regarded as medical devices. The study protocol was subsequently reviewed and approved by the Ethical Committee of the Faculty of Behavioural, Management and Social Sciences, University of Twente (file number 190141).

The current mixed-methods study consisted of standardised scoring of recorded patient consultations from MI-trained and non-MI-trained podiatrists and semi-structured in-depth interviews with the MI-trained podiatrists. The quantitative component consisted of audio recorded clinical consultations to measure the application of MI-skills in MI-trained podiatrists in comparison with non-MI-trained podiatrists. This was scored with the Motivational Interviewing Treatment Integrity (MITI) code [45]. The qualitative component consisted of in-depth interviews with the MI-trained podiatrists regarding their attitudes and experiences towards the use of MI and the implementation of the MI-techniques in daily clinical practice. This mixed-methods study design was chosen to obtain outcomes from different perspectives and contextualise the results of the MI-training [46]. Therefore, the results of the quantitative and qualitative components were combined through triangulation to integrate results, to come to a deeper understanding of both qualitative and quantitative results [47].

### Participants

The participants in this study were employed podiatrists at “Voetencentrum Wender”, which is a health organisation in the Netherlands, among others, providing treatment for people with diabetes foot disease. Only podiatrists located in the East of the Netherlands and treating one or more patients who participated in the RCT were included in the current study. The description of the stratified randomisation process of the podiatrists in the RCT is described in detail elsewhere [44]. Each participant in the RCT has been treated by their own MI-trained or non-MI-trained podiatrist or one of the other podiatrists from the same group (MI-trained or non-MI-trained) during the one-year follow-up.

Because in the RCT the ratio between patients in the intervention and control group became unbalanced, more podiatrists had to be trained than initially scheduled. This has also resulted in a skewed ratio between the number of podiatrists in the control and intervention group of the current study. This was due to the fact that one of the non-MI-trained podiatrists treated 37.7% (*N* = 26) of the patients in the control group. All podiatrists provided written informed consent to participate in the study.

### Intervention

An MI-trainer, affiliated with MINT (Motivational Interviewing Network of Trainers), trained two groups of podiatrists in MI during a three-day (21 h) basic training. The first group of podiatrists (*N* = 7) also received a one-day (7 h) online booster training a year after the face-to-face basic training. The second group of podiatrists (*N* = 11) received only a three-day (21 h) basic training online (due to COVID-19 restrictions). The podiatrists were trained to incorporate the specific coaching and communication techniques of MI in their consultations with the aim to increase adherence to wearing orthopaedic shoes in people with diabetes foot disease.

During the basic training, MI and the four processes of MI were explained, and different MI-techniques were discussed and practiced. The MI-techniques that were discussed are: asking Open questions, Affirmation, Reflective listening, and Summarising (OARS). These techniques were combined with giving information and advice with permission, how to elicit and strengthen change talk, handling ambivalence, softening sustain talk and reacting to discord [48]. The training varied in the mode of instruction including videos, theory and roleplay exercises with feedback. The training consisted of two consecutive days and a third training day after 10 days. In this way the podiatrists could become familiar with the principles of MI during the first two days and could use the learned MI-techniques directly in their consultations with their patients. On the third training day, there was a discussion about their practice experiences of applying MI. Based on a consultation of one of the podiatrists, that was audiotaped in clinical practice, in the training group they reflected on what went well and identified improvements. This learning process was supplemented with additional exercises and theory to strengthen and consolidate the experiences already gained.

Previous research demonstrated that the training effects might diminish quickly [25, 39, 40] and changes in communication style might not be maintained. Therefore, the MI-trainer sent monthly emails to encourage the podiatrists to keep using MI. In every email a short piece of theory was repeated, and an example of what the podiatrists could do to continue their MI approach was provided.

### Quantitative measures

To systematically observe and rate the application of MI, also called MI-fidelity, in the daily clinical practice of podiatrists, audio recordings were scored with the Motivational Interviewing Treatment Integrity 4.2.1. (MITI 4.2.1) coding system [49]. After the first training was completed the MI-trained and non-MI-trained podiatrists were asked to audio record at least one consultation with their patients. The researchers choose which consultations were to be audio recorded. Besides this, the consultations carried out by the MI-trained and non-MI-trained podiatrists had the same length. The MITI 4.2.1. is a reliable behavioural coding system that assesses which MI-related skills are applied during interactions, also called treatment fidelity, by coding the verbal communication behaviours of care professionals [49]. The MITI 4.2.1. consists of two components: global scores and behaviour counts. The global scores include a relational (partnership, empathy) and a technical (cultivating change talk, softening sustain talk) component to assess the use of MI. These were scored on a Likert scale from 1 (low) to 5 (high). In addition, fidelity was measured by counting ten behaviours: three main MI-adherent behaviours (affirmation, seeking collaboration, emphasising autonomy), two non-MI-adherent behaviours (persuading and confronting) and five other relevant behaviours (giving information, persuasion with permission, questions, simple and complex reflections). See ‘Additional file 1’ for a summary description of the MITI codes. With these scores the summary scores were calculated and compared with previously published, expert-derived “fair” thresholds. These “fair” thresholds, a beginner proficiency level, were considered the minimum extent of MI-application needed to obtain the desired behaviour change effects on clients [49]:

1. The relational score is the average of the partnership and empathy global scores. The “fair” threshold is set at 3.5. Higher scores indicate podiatrists foster a more collaborative approach and genuinely seek to understand a patient’s perspective.
2. The technical score is the average of the softening sustain talk and cultivating change talk global scores. The “fair” threshold is set at 3. Higher scores indicate podiatrists actively eliciting the patient’s arguments in favour of positive change and softening the patient’s sustain talk.
3. The reflection to question ratio is calculated, with the “fair” threshold set at one reflection to one question. Higher scores indicate that the podiatrist focuses on evocation and engagement.
4. The percentage of complex reflections is compared to the sum of complex and simple reflections. The “fair” threshold is set as ≥ 40%.

The coding was performed independently by an experienced MINT coder (JdJ) and a senior researcher (AB) who had been trained and supervised by the MINT coder (JdJ). Both coders were blinded to the MI-training status of the podiatrists. The interrater agreement for MITI coding was assessed on five randomly chosen recordings (20% of total recordings) between the two coders based on the intraclass correlation coefficients (ICCs). The ICCs were calculated using two way mixed effect models for absolute agreement of average measures [50]. The mean (± SD) interrater agreement between two coders was good (ICC = 0.70 ± 0.16). All intraclass correlation coefficients ranged between good for ‘affirmations’ (ICC = 0.62) and excellent for ‘giving information’ (ICC = 0.86), but only fair for ‘persuade’ (ICC = 0.56) and complex reflections (ICC = 0.48), and even poor for the behavioural count confront (ICC = 0.37) [51]. Due to lack of variance among the scores of the recording ratings, the ICCs for the global score on ‘softening sustain talk’ and the behavioural count on ‘seeking collaboration’ and ‘emphasising autonomy’ could not be calculated. For the five recordings that were scored by both coders, the average of both raters’ scores was calculated. The other recordings were scored by only one of the coders.

### Qualitative measures

Individual semi-structured in-depth interviews with the MI-trained podiatrists were conducted and recorded online via Microsoft Teams (Version 1.4, Microsoft, 2021) by two of the authors (BB and MJW) between mid-January and early February 2021. This was after the last patient was included in the RCT and respectively 9–19 months after the basic training of the second and first group of podiatrists. The following topics were discussed during the interview (see ‘Additional file 2’): podiatrists’ attitudes and experiences towards the use of MI and the implementation of the MI-techniques in daily clinical practice with people with diabetes at high-risk of foot ulcers (questions 7–10).

### Data analyses

Demographic data of the podiatrists and quantitative data of the MITI coding were analysed using IBM SPSS Statistics 27.0 (IBM, New York, USA). For the qualitative data analyses the research software ATLAS.ti 8.4 (Scientific Software Development GmbH) was used.

First, the two MI-trained groups of podiatrists were compared to each other, to make sure that they did not differ significantly from each other. Since they did not (*p* = 0.364–0.944) both groups were taken together for further analyses. Thereafter differences in demographics and quantitative data between independent groups for nominal data were assessed with Pearson chi-square tests. Differences in dependent ordinal data and continuous variables (due to skewed distributions) were assessed with Mann–Whitney U tests. A significance level of *p* < 0.05 was adopted for all statistical tests.

To explore whether there was a difference in communication style between the MI-trained and non-MI-trained podiatrists in the use of MI-related skills, consultations were scored from both groups of podiatrists. Only the consultations with a duration of 15 min or longer were coded according to the MITI guidelines [49]. The median of the coded consultation length was 20 min (range: 15–20). For the consultations with a duration of less than 20 min the whole consultation was coded and for the consultations longer than 20 min, a 20-min segment was coded, starting at 1.30 min until 21.30 min [49]. The first recorded consultation of each podiatrist was used in the analysis, unless the second recorded consultation was the one that was scored by both coders. This was the case in one of the five double coded recordings. The means, standard deviations, and ranges for the behaviour counts and summary scores across podiatrists, were calculated per group. The number of interactions reaching the “fair” threshold was counted for each consultation.

The in-depth interviews were conducted with 17 instead of 18 MI-trained podiatrists, because one of the podiatrists was no longer employed at the company. The audio recordings of the qualitative interview data of the MI-trained podiatrists were transcribed verbatim. After transcription, thematic analysis was conducted [52]. This procedure started with familiarisation of the data and generation of the initial codes. Then, the codes were transformed into topics and subtopics. The initial codes, topics and subtopics were identified by one of the authors (MJW) and discussed with two other authors (BB and CB). This was repeated until no new codes emerged. A second assessor (BB) assigned codes independently to 41 quotations, 10% of the total quotations. Thereafter, the interrater agreement (59%) was calculated, and the two assessors discussed any coding differences until consensus was reached and all interviews were checked to apply the consensus coding. The code scheme was developed by combining inductive and deductive thematic analysis [53] and can be found in ‘Additional file 3’. The main topics were set a priori by the researchers in the semi-structured in-depth interviews and the subtopics represent the content mentioned by the podiatrists during the interviews. The results of the interviews are structured according to the relational and technical components of MI to allow triangulation of the qualitative and quantitative results.

## Results

During the study 22 podiatrists treated one or more persons from the RCT who experienced diabetes and were at high-risk of developing foot ulcers. Eighteen podiatrists followed the three-day basic training or the three-day basic and one-day booster training, and four podiatrists were not trained in MI (Table 1). Participating podiatrists were aged between 25 and 51 years (median 29.5), 12 (54.5%) were female, and experience as podiatrist ranged from 2 to 26 years (median 7.00). Non-MI-trained podiatrists were only women (*p* = 0.044) and had more years of experience (*p* = 0.039) than the MI-trained podiatrists. Seven podiatrists, five of the MI-trained and two of the non-MI-trained podiatrists, had little MI knowledge before the start of the study via a course/lecture during their podiatry training or through self-study.

**Table 1:** Demographic data of the podiatrists

	**MI-trained podiatrists (*N* = 18)**	**Non-MI-trained podiatrists (*N* = 4)**	***P*-values**
Age (median (y), IQR)	28.5 (26–34.75)	38.5 (31.5–47.5)	0.060
Gender (M/F)	10/8	0/4	0.044 ^*^
Experience as podiatrist (median (y), IQR)	4.5 (2.5–10.75)	14.75 (8.00–23.25)	0.039 ^*^
Experiences with MI			0.706
Unknown	1 (5.6%)	-	
Unfamiliar with MI	3 (16.7%)	-	
Familiar with the name MI	9 (50.0%)	2 (50.0%)	
MI knowledge	5 (27.8%)	2 (50.0%)	

Note: *F* female, *IQR* interquartile range, *M* male, *MI* motivational interviewing, *N* number, *y* year. Percentages may not add up to 100 due to rounding

^*^ Significantly difference, *p* < 0.05

### MITI results

Fourteen audio recordings from the MI-trained and four audio recordings from the non-MI-trained podiatrists were 15 min or longer and were rated with the MITI. These recorded consultations occurred between 6 and 22 months after following the MI-training.

Two of the four “global scores” (empathy and change talk), one of the five core “behaviour counts” (affirm) and one of the other behaviour counts (simple reflections) were rated significantly higher for the MI-trained podiatrists (Table 2). Comparing the results of the four MITI summary scores with the “beginner proficiency level” thresholds [49], one MI-trained podiatrist met all four thresholds, four MI-trained podiatrists met three thresholds and also four MI-trained podiatrists met two thresholds (Table 3). Two MI-trained podiatrists and two of the non-MI-trained podiatrists met none of the four thresholds.

**Table 2:** MITI coding results of audiotaped interactions of MI-trained (*N* = 14) and non-MI-trained podiatrists *(N* = 4)

MITI variable	MI-trained Podiatrists Mean (SD; Range)	Non-MI-trained Podiatrists Mean (SD; Range)	*P*-values
**Global scores – Relational**			
Partnership	2.71 (0.70; 1.50–4.00)	2.13 (0.25; 2.00–2.50)	0.114
Empathy	3.57 (0.78; 2.00–5.00)	1.75 (0.96; 1.00–3.00)	0.008 ^*^
**Global scores – Technical**			
Change talk	3.18 (1.03; 1.00–5.00)	1.25 (0.50; 1.00–2.00)	0.008 ^*^
Soften sustain	3.04 (0.57; 2.00–4.00)	3.00 (0.00; 3.00–3.00)	0.788
**Behaviour counts**			
Questions	13.75 (7.68; 4.00–31.00)	8.75 (6.40; 0.00–14.00)	0.489
Simple Reflection	9.11 (5.60; 2.00–21.00)	1.38 (1.49; 0.00–3.50)	0.008 ^*^
Complex Reflection	3.00 (2.65; 0.00–9.00)	1.50 (1.68; 0.50–4.00)	0.179
Giving Information^**†**^	2.86 (2.11; 0.00–7.00)	5.00 (7.44; 0.00-–6.00)	0.788
Persuade with Permission	0.57 (0.94; 0.00–2.00)	0.25 (0.50; 0.00–1.00)	0.684
***MI-adherent behaviour***			
Affirm	3.75 (3.33; 0.00–12.00)	0.63 (0.95; 0.00–2.00)	0.036 ^*^
Seeking Collaboration	0.57 (0.85; 0.00–3.00)	0.25 (0.50; 0.00–1.00)	0.496
Emphasising Autonomy	0.11 (0.30; 0.00–1.00)	0.25 (0.50; 0.00–1.00)	0.567
MI-adherent behaviour total	4.43 (3.94; 0.00–15.00)	1.13 (1.93;0.00–4.00)	0.076
***MI-non-adherent behaviour***			
Persuade^**†**^	3.36 (2.73; 0.00–8.00)	1.38 (1.25; 0.00–3.00)	0.197
Confront	0.50 (0.96; 0.00–3.50)	0.13 (0.25; 0.00–0.50)	0.526
MI-non-adherent behaviour total	3.86 (3.49;0.00–11.50)	1.50 (1.29; 0.00–3.00)	0.216
**MITI summary scores**			
Relational score	3.14 (0.71; 2.00–4.50)	1.94 (0.43; 1.50–2.50)	0.009 ^*^
Technical score	3.11 (0.66; 1.50–4.50)	2.13 (0.25; 2.00–2.50)	0.011 ^*^
Reflection to question ratio	1.02 (0.64; 0.10–2.83)	0.29 (0.10; 0.19–0.38)	0.023 ^*^
Percentage complex reflections	23.42 (15.35; 0.00–57.14)	56.46 (40.49; 12.50–100.00)	0.136

Note: *SD* standard deviation

^*^ Significantly different between groups, *p* < 0.05

^§^ Lower Persuade or Giving Information scores indicate better MI-adherence

**Table 3:** MITI summary scores of audiotaped interactions of MI-trained (*N* = 14) and non-MI-trained podiatrists (*N* = 4)

MITI summary scores	Threshold ^§^	Threshold reached N (%)	MI-trained/Non-MI-trained	*P*-values
Relational score	≥ 3.50	8 (44)	8/0	0.043 ^*^
Technical score	≥ 3.00	11 (61)	11/0	0.004 ^*^
Reflection to question ratio	≥ 1.00	7 (39)	7/0	0.070
Percentage complex reflections	≥ 40	4 (22)	2/2	0.130

Note: ^*^Significantly different between groups, *p* < 0.05

^§^The fair threshold was used [49]

### Interview results

The interviews had a median interview length of 17 min (range 13–32 min). The interview results can be found below described in the five main topics. From the interviews 28 subtopics were identified (see ‘Additional file 3’). Only quotes with rich value for understanding of the sub codes are mentioned below. A complete list with all quotes belonging to each subtopic is provided in ‘Additional file 4’.

#### Main topic 1. Podiatrists’ perspective regarding the goal of MI

The podiatrists indicated partnership (relational component of MI), cultivating change talk (technical component of MI) and motivating patients as the goals of MI. They thought that MI helped them to speak with patients on an equal level and gave them the possibility to achieve a specific goal together with the patient. Besides this, MI enabled them to encourage patients to think about their own perspectives.

*“That you make the patient think about why something might (not) work for him/her and very often then they come to new insights”* (Pod07)

MI moreover addressed the intrinsic motivation of patients to change their behaviour.

*“The goal of MI is to activate people from within themselves to apply to something, as in this study a certain therapy. So it is not something that is imposed by us, but that they understand themselves why it is necessary and that it comes from themselves, intrinsic motivation”* (Pod02)

Additionally, podiatrists indicated that MI involves using other communication techniques, such as reflective listening, asking reflective questions and softening sustain talk.

#### Main topic 2. Experiences related to MI-training

##### Subtopic 2.1. New insights

During and after the MI-training, the podiatrists gained new insights. They indicated they had learned that partnership (relational component of MI) means to speak with patients on an equal level, that it is important to reflect on patients’ ideas, and that their task is broader than only providing information. The podiatrists also realised that it is not beneficial to persuade without the patient’s permission, that it is important to express fewer prejudices towards the patient and that they should try to avoid conflict in their working alliance with the patient. In addition, the podiatrists also gained new insight regarding cultivating change talk (aspect belonging to the technical component of MI). Changing the podiatrists’ communication style helped patients to think from their own perspective. With regards to the other MI-techniques the podiatrists learned that asking open questions instead of closed ones led to more insight into the patients’ motives and needs, and that using silences could be useful to let patients think from their own perspective instead of overwhelming them with expertise-based advice.

##### Subtopic 2.2. Behavioural change for podiatrist

Some of the podiatrists realised that the use of the MI-techniques will be a substantial behaviour change for themselves, because they recognised that their traditional communication techniques were (very) different from the MI-techniques.

*"It really made me realise that I was used to use such a different [traditional] communication technique during the last years, and it also made me realise that it is also a very substantial adjustment for me to change that”* (Pod04)

##### Subtopic 2.3. Applicability of MI

Other podiatrists indicated that the MI-techniques would easily be applicable since their usual communication techniques were similar to those used in MI. Also, the practice-oriented approach and the use of many examples during the MI-training made applying MI in practice easy for the podiatrists.

*“I found out that I actually already unconsciously applied certain things in practice in the same way. That's all named as motivational interviewing. I thought that sounds very familiar to me...It was nice to hear that you actually already did something and they tell you how to do it. That you think: I actually already did that unconsciously”* (Pod01)

Nevertheless, some podiatrists indicated that as point for improvement it would even be better to match the MI-training content more closely to the specific target groups, the examples given should be more related to the users and recipients of MI. A second point for improvement, most podiatrists reported that they would like to have feedback regarding their application of MI, so it is clear to them whether they apply the MI-techniques correctly in practice.

##### Subtopic 2.4. Multimodal training method

With regards to the multimodal training method, podiatrists experienced alternating between listening, interaction with the trainer, and exercises with each other during the MI-training, and the small training group pleasant. They also valued that the trainer was able to tailor the MI-training content to their knowledge. Besides these positive experiences, some podiatrists had also some points for improvement and suggested that the experience of the MI-training would have been better if it had been possible to meet physically instead of video conferencing (due to COVID-19 restrictions), and they felt that the quantity of information supplied was too much for the relatively short training time.

##### Subtopic 2.5. Importance of repeating MI-training information

Repetition of the (content of) MI-trainings was indicated as important. It was particularly useful for the podiatrists to receive the monthly emails, and to have the one-day booster training to refresh their knowledge and remind them to consciously apply MI in practice. However, as a point for improvement, the podiatrists mentioned that more repetition of the (content of) MI-trainings was necessary so to become familiar with using MI in daily clinical practice.

#### Main topic 3. Podiatrists’ experiences with MI in practice

##### Subtopic 3.1. Partnership

Within the relational component of MI, the podiatrists experienced that partnership was normal to them because they were used to collaborating with the patient. The podiatrists reported that this partnership became easier due to thinking along and/or asking questions; that it ensured the podiatrist spoke with the patient on an equal level; that working together was easier with a motivated patient; and that the use of MI even led to better results of podiatry (less diabetic foot problems) or behavioural changes in patients.

MI-adherent behaviours like affirmation and seeking collaboration were experienced as necessary to keep a patient motivated. It was important to connect with the patient and not only to provide information. Other MI-techniques, such as giving information and persuasion with permission were mentioned as important because patients are not always familiar with the possible treatment options for their diabetic feet. However, the podiatrists realised that they needed permission to persuade, otherwise patients would probably not show a behavioural change. Yet there was one podiatrist who found it difficult to stop automatic repair and advice reflexes which means that the podiatrist tries to solve the problem for the patient.

##### Subtopic 3.2. Change talk

The experiences of the podiatrists differed regarding cultivating change talk within the technical component of MI. Some podiatrists mentioned positive experiences, e.g., that the use of MI by the cultivation of change talk made patients think from their own perspective and that it provided in-depth conversations between the podiatrist and patient. On the other hand, several podiatrists reported negative experiences, e.g., they experienced this technique as difficult because it was novel for them and therefore was a point for self-. Change talk was also experienced as difficult by the podiatrists, because some patients in this patient group were not always familiar with the treatment options for diabetes foot disease.

##### Subtopic 3.3. Acceptance

Within the relational component empathy, the podiatrists had different experiences with acceptance of the patient’s choice, opinion and/or behaviour. They thought it was natural to accept the patients’ choices, opinions, and/or behaviours, and they experienced that by accepting this the podiatrist was letting the patients think for themselves. However, the podiatrists realised that they also needed to give the patients time to let them think for themselves about possible changes.

*“And if it really doesn’t work right away, then I’ll just take a little longer and let the patient come back sometime or give them more time to think about it…The more compelling I come across, or the more I demand of the patient, the greater the patient’s shield becomes against me, so therefore I give people a little more rest and time [to think]”* (Pod16)

Besides this, the podiatrists also mentioned that it can be difficult to accept the wishes of non-cooperative patients.

##### Subtopic 3.4. Compassion

The podiatrists mentioned that they did not experience any problems with compassion within the relational component empathy, because they thought that helping others without benefiting themselves belonged to their mindset towards patients as a healthcare provider.

##### Subtopic 3.5. Ask open questions

Regarding the MI-technique open questions, the podiatrists’ experiences differed. Some podiatrists mentioned that they found it easy to ask open questions, while others experienced more difficulty in asking open questions than expected, particularly asking about the reason why a patient did not want to change.

*“Especially asking questions, asking open questions is more difficult than I thought, because you actually think you always ask open questions, but you actually ask much more closed questions [than you think]. And if you have someone who is very closed off and you ask closed questions, you actually get very little information”* (Pod03)

##### Subtopic 3.6. Applicability of MI

The podiatrists had different experiences regarding the applicability of MI. Some podiatrists reported that they experienced no problems changing from their usual approach to the MI-related communication techniques. This was because these communication techniques were similar to their own techniques. Conversely, some other podiatrists mentioned that applying the tips and tricks of the MI-training made the use of MI feel unnatural and uncomfortable, because the podiatrist had to ask the patient more questions than usual. However, the application of MI during a foot examination made the use of MI feel more natural.

There were also other reasons why the podiatrists experienced difficulties using MI. A couple of them mentioned that it was difficult because there were other matters that had to be discussed during an appointment, and also due to their own working method. Furthermore, the podiatrist’s empathy of the patient’s situation made it difficult to continue using the MI-techniques, as evidenced by Pod04 comment:

*“For example, there is a home situation in which people very quickly say ‘I’ll take my shoes off’. I find it very difficult to motivate those people, because I understand why those people take their shoes off” (Pod04)*

There were some podiatrists who reported that the use of MI was difficult due to negative experiences and because other communication techniques seemed more effective to them. This created doubts about the applicability of MI in practice.

The podiatrists experienced that the application opportunities for MI depended on the characteristics of the patient and on the level of their familiarity with the patient. For example, the use of MI was easier with established patient relationships and more difficult with unknown patients. Some podiatrists mentioned that the use of MI was also experienced as difficult if the patient was not engaging, while others mentioned that a “challenging” patient encouraged them to apply MI.

*“With certain difficult patients where communication does not run completely smoothly, then you would rather think of applying MI. You think about, how can I collaborate with the patient, so that we can work together towards one goal”* (Pod13)

##### Subtopic 3.7. Behavioural change for podiatrist

Some podiatrists experienced the use of MI as development or even led to a behavioural change for themselves.

*“I’ve been working as a podiatrist for 10 years so you’re also completely set in your own ways and your own things…it is indeed a complete change, the use [of MI] itself is still quite difficult”* (Pod15)

Therefore, the podiatrists mentioned that they had not (yet) always applied MI in daily clinical practice, despite some of them being aware that the traditional communication techniques were no longer the solution. In general, the podiatrists realised that to ensure an integrated, fruitful, or smoother application of MI, that frequent use of MI was required. This would be necessary because there is a risk that information from the MI-training would become diluted or completely forgotten from usage. Some podiatrists thought that they applied MI already unconsciously, because they were already using it; others reported to be consciously engaged. In addition, not all podiatrists used the exact theoretical version of the MI-techniques as taught during the MI-training, but used the details that they thought they could apply to themselves.

##### Subtopic 3.8. Added value of MI

Many of the podiatrists believed MI was of added value, especially cultivating change talk, one of the technical components of MI. The use of MI helped to make patients think for themselves, to make conscious choices and even led to behavioural changes in patients. In addition, it was reported that cultivating change talk was especially of added value for podiatrists who had difficulty evocating a behavioural change in their patients.

The podiatrists also reported that the added value of MI depended on the characteristics of the patient, whereby MI was of added value for, e.g., non-adherent/uninformed/unmotivated patients. Besides this, they experienced that patients have to be open minded to MI in order for it to have added value and that MI had only an added value for patients with whom they had frequent contact.

##### Subtopic 3.9. Dealing with resistance to orthopaedic shoes

A combination of some of the MI-techniques were used by a few podiatrists to deal with resistance to orthopaedic shoes, including partnership, which is one of the relational MI components. In addition, the podiatrists accepted the patients’ resistance and informed patients about the treatment options for their foot disease. By informing patients about these unfamiliar possibilities, the podiatrist encouraged the patients to think for themselves.

#### Main topic 4. Patients’ experiences observed and mentioned by the podiatrist

In addition to their own experiences, the podiatrists were also asked about the observed experiences of the patients regarding the use of MI in their consultations. Related to partnership (relational component of MI) and cultivating change talk (technical component of MI) the podiatrists mentioned different observed patients’ experiences. Many podiatrists reported that the patients experienced working together with the podiatrist as pleasant. Besides this, a single podiatrist reported that some patients showed a more open attitude. However, they also mentioned that it took time for some patients to get used to working together with the podiatrist, because they were unfamiliar with this way of communicating with their podiatrist. They also observed that cultivating change talk made the patients realise that they themselves could contribute to behavioural change and made them see the importance of wearing orthopaedic shoes. However, it also gave the patient insight into their behaviour which was not always welcomed, because this was confronting for the patient. In addition, within the MI-adherent behaviours, the podiatrists mentioned that the confirmation from the podiatrist that things were going well was experienced as pleasant by the patient. Conversely, the podiatrists reported that some patients experienced the (open) questions in MI-style as unpleasant. Because of their digital patient reporting system the podiatrist already had to ask a lot of questions, and therefore in some cases the use of MI might not be applicable.

#### Main topic 5. Recommendations

Most podiatrists in this study recommended MI to all other podiatrists, where they emphasised partnership within the relational component of MI and cultivation of change talk within the technical component. This is because working together with the patients ensured that behavioural change could be reached through cultivating change talks, which made the patient think for them self. The podiatrists also reported other outcomes with the use of MI. It provided the podiatrist with some background knowledge about communication techniques and led to better conversations. MI also sensitises the podiatrist to quickly recognize whether a patient showed sustain talk or change talk.

Some podiatrists even recommended adding MI within the primary podiatry education, because this would ensure regular repetition of the content of MI-training. In addition, it also provided the podiatrist with insight into and allow them to focus on patients’ expectations and wishes from the beginning of their education. However, a couple of podiatrists recommended the use of MI, but had doubts about including MI in the podiatry training since it might be better to follow an MI-training once the podiatrists had obtained experience in practice.

### Data triangulation

The results of the quantitative and qualitative components were combined through triangulation to obtain outcomes from different perspectives and contextualise the results of the MI-training. The MI-trained podiatrists appeared to have acquired basic knowledge and skills regarding MI, but had not yet become MI-experts. The observed communication behaviours in the MITI-scored consultations showed that the podiatrists applied less complex MI-related skills with regard to the relational and technical components of MI that is supported by what they were able to mention during the interviews. The MI-trained podiatrists showed clearly better MITI results on partnership, empathy and cultivating change talk compared to the non-MI-trained podiatrists and demonstrated their understanding of partnership and cultivating change talk in the interviews. The acquired knowledge and skills enabled the communication between podiatrists and their patients in a collaborative and empathetic way, which stimulated behaviour change in the patient towards adherence with recommended foot self-care. However, more complex MI-related skills were minimally applied by the podiatrists in practice and were not mentioned in the interviews with the MI-trained podiatrists. One of those skills was applying complex reflections. The MITI results showed that the threshold for complex reflections was only achieved by two of the fourteen MI-trained podiatrists, compared to two of the four non-MI-trained podiatrists. However, the MI-trained podiatrists used many more reflections, both simple and complex, compared to the non-MI-trained podiatrists.

Most MITI results correspond well with the interview results, although one contradiction was found. The MITI results showed a difference between the MI-trained and non-MI-trained podiatrists on the MI non-adherent behaviour variable ‘persuade without permission’. Here, the non-MI-trained podiatrists tended to score better. Possibly, MI-trained podiatrists know that giving advice is allowed, but that they simply forgot to ask the patient for permission to give advice or to ask what the patient thinks of their advice. This means that the MI-trained podiatrists use the MI-techniques only partially and that the non-MI-trained podiatrists give less advice than the trained podiatrists. This is in line with the interview results, because only one MI-trained podiatrist realised that giving advice without permission may not lead to a behaviour change in the patient. Additionally, the qualitative results showed that the podiatrists experienced the use of MI as patient dependent, e.g., MI is more difficult to apply with an already motivated person or a person who is not open to it. This is also clearly seen in the MITI results per podiatrist. During one conversation with a patient, the podiatrist applied the basic principles of MI at a beginner’s level, while during another conversation the same podiatrist did not apply MI at all. However, the MITI results also showed a contradiction with the interview results. The fact that the MI-trained podiatrists scored significantly better on the relational component empathy than the non-MI-trained podiatrists was unexpected, because during the interviews the podiatrists indicated that compassion belongs to their mindset towards patients.

## Discussion

The current study aimed to analyse the MI-fidelity in consultations carried out by MI-trained and non-MI-trained podiatrists. It also explored podiatrists’ attitudes and experiences towards the implementation of the MI-techniques in practice. The main findings of this mixed methods study indicate after data triangulation that at 6 to 22 months after following the MI-training, the MI-trained podiatrists used the principles of MI at a solid beginner proficiency level, fair scores on the MITI, which is in accordance with the basic MI-training they received. As expected, MI-trained podiatrists did this significantly better than non-MI-trained podiatrists. The MI-trained podiatrists scored significantly better on partnership within the relational component of MI and cultivating change talk within the technical component of MI. These were also the specific MI-related skills that the podiatrists themselves described relating to their attitudes and experiences of using MI in practice. However, they are not able to reproduce all MI-related skills that have been taught. This corresponds to the podiatrists’ comments that they have learned and realised that MI is not a trick to be applied, but is a new communication technique to acquire and takes time to apply correctly and fully in practice.

The results of the data-triangulation of this study are in line with previous research on MI-training for diabetes healthcare providers. Two studies by Brug et al. and Welch et al. showed that facilitating change talk and asking open questions are the MI-related skills that most frequently improved following training [54, 55]. Kaczmarek and colleagues suggested that the reason for this may be that these skills are less complex to learn and easier to apply during clinical practice compared to some other MI-related skills [42]. In the studies by Brug et al. and Magill et al., empathy and the MI spirit were also increased [54, 56]. Other MI-related skills, for example complex reflections, seem to be more difficult to acquire and apply during clinical practice [25, 42]. Doherty and colleagues also noted that reduction of confrontation is experienced as a complex MI-related skill for diabetes healthcare providers [57]. The duration of the MI-training in the current study (21 h and 28 h) was similar to the duration of the MI-training in the studies included in the systematic review by Kaczmarek and colleagues [42], which ranged from 2–40 h. The training in the current study also consisted of didactic training in concepts of MI, role play, and video examples such as the MI-training in previous studies [42]. Like Kaczmarek et al., we can conclude that podiatrists can be trained in MI [25]. However, in contrast to their results that no improvements in MI-related skills remained after 12 weeks, the podiatrists in the current study still used the principle of MI at a solid beginner proficiency level 6–22 months after following the MI-training [25]. This may be due to the fact that the podiatrists in the current study had many more hours of MI-training than in the study of Kaczmarek et al. and that the podiatrists in the current study received monthly emails to support them to keep using MI in daily clinical practice [25]. It might be seen as a limitation of this study that also in the non-MI-trained group two podiatrists have heard about MI and two others described themselves as having MI knowledge, the results showed that only substantial training in MI helps podiatrists to implement this communication approach reliable in their clinical practice.

Regarding the implementation of MI in practice, the current study provides some recommendations. First, we recommend that the digital patient reporting system should be adapted in such a way the podiatrists can integrate MI-techniques more easily into their work. The use and maintenance of new skills in routine practice should be facilitated and not hindered by contextual factors as the current reporting system [58, 59], as also mentioned by the podiatrists. The digital patient reporting system they use already requires to ask many questions to complete a patient file. This might lead to an overload of topics to discuss with a patient during one consultation when implementing MI. Second, we recommend including MI within the primary podiatry education. This will promote a successful use of MI in practice, because of regular repetition of the content of the MI-training and the provision of frequent feedback. This prevents the podiatrist from reverting to their usual communication techniques. In addition, the inclusion of MI in the podiatry education from the beginning of their education provides the podiatrist with insight into and focus on patients’ expectations and wishes.

A strength of this study is the mixed-method approach with data triangulation, providing more robust evidence than in the previous pilot studies [25, 43]. Further, the audio recorded consultations carried out by the MI-trained and non-MI-trained podiatrists were assessed by two independent external coders who were blinded to the MI-training status of the podiatrists. Finally, the coders only counted the behaviours of the podiatrists on relevant aspects of the consultations, so the behaviours of the podiatrists during off-topic speaking were not counted [60].

The results of this study may be limited by the following. Firstly, by the skewed ratio between the number of podiatrists in the intervention and in the control group, which means that the results of this study must be interpreted with caution. This skewed distribution was unavoidable, because the current study was part of the RCT which took place in clinical practice [44]. The purpose of the RCT was to investigate the good clinical use of medical resources, in this case orthopaedic shoes. The importance of highly external valid outcomes overrode aspects of internal validity, such as normal distribution of the podiatrists. However, this high external validity makes the results of the study applicable for education and training purposes in practice [61]. Besides, doing research in clinical settings with vulnerable patients requires flexibility in study design. Therefore, we think the mixed-methods study design is very helpful to collect data in a concise way. Secondly, the mean ICC levels for persuade and complex reflection were only fair and, even poor for the behavioural count confront [51], despite both coders agreement on the description of the MITI codes, which are standardised and valid [49]. There can be different causes of a low ICC. This low ICC can reflect the low degree of rater agreement but might also be related to the lack of variability among the sampled subjects, the small number of subjects, and the small number of raters being tested [62, 63]. In the current study within the coding of reflections one of the coders seemed to be stricter in assigning a complex reflections than the other. This affected the results of the percentage complex reflection, one of the MITI summary scores, which means that the podiatrists rated by this coder was less likely to reach the “fair” threshold. Within the behavioural count confront a small difference in coding between both coders had a big impact on the ICC level of this code, because it was only applied a few times by the podiatrists. Therefore it would be better to have more than two coders or have all recordings rated by both coders. In addition, due to lack of variance, the ICCs for the global score on softening sustain talk and the behavioural count on seeking collaboration and emphasising autonomy could not be calculated at all. For softening sustain talk this may be since the description of this MITI codes was so clear for both coders that the variance between them was small or none. Besides this, when seeking collaboration and emphasising autonomy this lack of variance can possibly be caused due to the podiatrists hardly applying these behaviours. These are more complex MI-related skills that may require more training to apply in daily clinical practice.

## Conclusion

Following the triangulation of the qualitative and quantitative results it can be concluded that after a basic MI-training, podiatrists can be effectively applying MI in daily clinical practice at a solid beginner level, with fair scores on the MITI. Furthermore, the findings of the current study support implementation of MI in practice but encourage MI training in the primary podiatrist training and maintenance training for daily clinical practice.

## Supplementary Information

**Additional file 1.** Summary description of MotivationalInterviewing Treatment Integrity (MITI) codes

**Additional file 2.** Overview of the main questions of the in-depthinterview

**Additional file 3.** Main topics based on the interview outcomes

**Additional file 4.** Supplementary quotes – (Pod = Podiatrist)

## Data Availability

The datasets used and/or analysed during the current study are available from the corresponding author on reasonable request after publication of the results.

## Abbreviations

BMS: Behavioural, Management and Social sciences
CE: Conformité Européenne
CMO: Commissie Mensgebonden Onderzoek
GmbH: Gesellschaft mit beschränkter Haftung
ICC: Intraclass Correlation Coefficients
MDR: Medical Device Regulation
MI: Motivational Interviewing
MINT: Motivational Interviewing Network of Trainers
MITI: Motivational Interviewing Treatment Integrity
OARS: Open questions, Affirmation, Reflective listening, and Summarising
Pod: Podiatrist
RCT: Randomised Controlled Trial
SD: Standard Deviation
WMO: Wet Maatschappelijke Ondersteuning
