# Supplementary material for: Attitudes and experiences towards the application of motivational interviewing by podiatrists working with people with diabetes at high-risk of developing foot ulcers: a mixed-methods study
Source: J Foot Ankle Res. 2022 Aug 19;15:62. doi: 10.1186/s13047-022-00567-y (PMC9388362; doi:10.1186/s13047-022-00567-y)
Supplement: Supplementary file 1 — Additional file 1. Summary description of MotivationalInterviewing Treatment Integrity (MITI) codes [file 13047_2022_567_MOESM1_ESM.docx]

| **Additional file 1: Summary description of Motivational Interviewing Treatment Integrity (MITI) codes** |
| --- |

| MITI codes | Short description |
| --- | --- |
| Global scores - Relational |  |
| Partnership | How well is the podiatrist in sharing power with the patient and stimulating equal participation during the conversation. |
| Empathy | How well does the podiatrist appear to achieve a deeper understanding of the patient during the conversation. |
| Global scores - Technical |  |
| Cultivating change talk | How well is the podiatrist working on evoking and cultivating change talk during the conversation. |
| Soften sustain talk | How well is the podiatrist working on softening and leading attention away from sustain talk during the conversation. |
| Behaviour counts |  |
| Questions | The podiatrist asks the patient a question. |
| Simple Reflection | The podiatrist reflects solely on what the patient has said. |
| Complex Reflection | The podiatrist reflects beyond what the patient has said, introducing new meaning and direction. |
| Giving Information | The podiatrist shares information, with attempting to persuade. |
| Persuade with Permission | The podiatrist asks for permission before advising or informing the patient on how to change behaviour. |
| *MI adherent behaviour* |  |
| Affirm | The podiatrist positively affirms the patient’s behaviour, intentions or strengths. |
| Seeking Collaboration | The podiatrist asks for permission to share information or advice, sharing power with the patient. |
| Emphasising Autonomy | The podiatrist states that the patient has the freedom to make his/her own decisions. |
| *MI non-adherent behaviour* |  |
| Persuade | The podiatrist gives unsolicited advice and persuades the patient to change behaviour. |
| Confront | The podiatrist argues, blames, judges or moralised the patient’s behaviour or decisions. |
