# Supplementary material for: Attitudes and experiences towards the application of motivational interviewing by podiatrists working with people with diabetes at high-risk of developing foot ulcers: a mixed-methods study
Source: J Foot Ankle Res. 2022 Aug 19;15:62. doi: 10.1186/s13047-022-00567-y (PMC9388362; doi:10.1186/s13047-022-00567-y)
Supplement: Supplementary file 2 — Additional file 2. Overview of the main questions of the in-depthinterview [file 13047_2022_567_MOESM2_ESM.docx]

| **Additional file 2: Overview of the main questions of the in-depth interview** |
| --- |

| Interviewer Question |
| --- |
| 1. Can you explain the purpose of motivational interviewing? (in your own words) |
| 2. What has it meant for you to apply motivational interviewing? |
| 3. How did you experience the MI-training? (0 = not useful at all and 10 = very useful) |
| 4. Has the MI-training led to new insights for you? (0 = no new insights at all and 10 = a lot of new insights) |
| 5. How did you experience the change from your usual patient approach to applying motivational interviewing? |
| 6. How did you experience communicating with the patient taking into account the basic principles of MI (partnership, evocation, acceptance and compassion)? |
| 7. How do you think your patients experienced your use of MI? (0 = very negative and 10 = very positive) |
| 8. Is the use of MI seen by the patient as an added value? |
| 9. In your opinion, is MI of added value in this group of patients compared to your normal patient approach? |
| 10. Have you encountered resistance from your patients with regard to wearing orthopaedic shoes? If so, how did you deal with this? |
| 11. Would you recommend applying MI to all podiatrists? In which way do you recommend the use of MI to all podiatrists? |
| 12. Did you miss something with regard to the MI-training and/or in the period after the training? What would you advise to improve in the future? |
