# Supplementary material for: Attitudes and experiences towards the application of motivational interviewing by podiatrists working with people with diabetes at high-risk of developing foot ulcers: a mixed-methods study
Source: J Foot Ankle Res. 2022 Aug 19;15:62. doi: 10.1186/s13047-022-00567-y (PMC9388362; doi:10.1186/s13047-022-00567-y)
Supplement: Supplementary file 3 — Additional file 3. Main topics based on the interview outcomes [file 13047_2022_567_MOESM3_ESM.docx]

| **Additional file 3: Main topics based on the interview outcomes** |
| --- |

| Main topic | Subtopics | |
| --- | --- | --- |
| 1. Podiatrists’ perspective with regard to the goal of MI | 1.1. Partnership |  |
|  | 1.2. Change talk |  |
|  | 1.3. Motivating by podiatrist |  |
| 2. Experiences related to MI-training | 2.1. New insights | 2.1.1. Partnership |
|  |  | 2.1.2. Change talk |
|  |  | 2.1.3. Ask open questions |
|  |  | 2.1.4. Allow for silences |
|  | 2.2. Behavioural change for podiatrist |  |
|  | 2.3. Applicability of MI |  |
|  | 2.4. Multimodal training method |  |
|  | 2.5. Importance of repeating MI-training information |  |
|  | 2.6. Points of improvement | 2.6.1. Applicability of MI |
|  |  | 2.6.2. Multimodal training method |
|  |  | 2.6.3. Importance of repeating MI-training information |
| 3. Podiatrists’ experiences with MI in practice | 3.1. Partnership |  |
|  | Dea3.2. Change talk |  |
|  | 3.3. Acceptance |  |
|  | 3.4. Compassion |  |
|  | 3.5. Ask open questions |  |
|  | 3.6. Applicability of MI |  |
|  | 3.7. Behavioural change for podiatrist |  |
|  | 3.8. Added value of MI | 3.8.1. Change talk |
|  |  | 3.8.2. Patient dependent |
|  | 3.9. Dealing with resistance to orthopaedic shoes | 3.9.1. Partnership |
| 4. Patients’ experiences observed and mentioned by the podiatrist | 4.1. Partnership |  |
|  | 4.2. Change talk |  |
| 5. Recommendations | 5.1. Application MI by all podiatrists | 5.1.1. Partnership |
|  |  | 5.1.2. Change talk |
|  | 5.2. Include MI in the primary podiatry education |  |
