# Supplementary material for: Attitudes and experiences towards the application of motivational interviewing by podiatrists working with people with diabetes at high-risk of developing foot ulcers: a mixed-methods study
Source: J Foot Ankle Res. 2022 Aug 19;15:62. doi: 10.1186/s13047-022-00567-y (PMC9388362; doi:10.1186/s13047-022-00567-y)
Supplement: Supplementary file 4 — Additional file 4. Supplementary quotes – (Pod = Podiatrist) [file 13047_2022_567_MOESM4_ESM.docx]

| **Additional file 4: Supplementary quotes – (Pod = Podiatrist)** | |
| --- | --- |
| 1. **Podiatrist’s vision with regard to the goal of MI** | |
| - 1. **Partnership** | "So, I actually think that there is much more communication on an equal level with the patients about the possibilities and what they prefer to do, to ensure that it [the wound] closes and they won't have complaints anymore” (Pod09) |
| - 1. **Change talk** | “That you make the patient think about why something might (not) work for him/her and very often than they come to new insights” (Pod07)  “You do not impose anything on people, but you actually address the intrinsic motivation of the patient” (Pod17) |
| - 1. **Motivating by podiatrist** | “Motivate your patients or clients to do something through communication techniques” (Pod06) |
| 1. **Experiences related to MI-training** | |
| - 1. **New insights** | |
| - - 1. Partnership | “Speak with the patient on an equal level” (Pod09)  “Let much more [information] come from your patient instead of working from the offering role” (Pod02)  “That you really like to throw your advice on the table, and without that man or woman perhaps being open to it” (Pod11)  “…What I actually learned through the exercises that we did during the courses, that I sometimes have to express my prejudices a little less quickly…” (Pod14)  “That you have to try to stay out of conflict with your patient, that’s just clearly explained in this course” (Pod14) |
| - - 1. Change talk | “…that a patient should actually give the answer himself, or should think for himself… That you really must have the reaction of the patient in order to be able to take a step towards behavioural change” (Pod05) |
| - - 1. Ask open questions | “…you very quickly ask closed questions, and then you do not always get the correct information or underlying information. So I also learned to ask questions differently so that they [the questions] become open and patients also have to tell a bit more” (Pod16) |
| - - 1. Allow silences | “That allowing silences can be very useful every now and then” (Pod02) |
| - 1. **Behavioural change for podiatrist** | "It really made me realise that I already use/used such a different communication technique during the last years, and it also made me realise that it is also a very substantial adjustment for me to change that” (Pod04) |
| - 1. **Applicability of MI** | “I found out that I actually already unconsciously applied certain things in practice in the same way. That's all told as motivational interviewing. I thought that sounds very familiar to me...It was nice to hear that you actually already did something and they tell you how to do it. That you think: I actually already did that unconsciously” (Pod01)  "Super practical, very good practical examples, you can immediately start using it [MI] the day after [the training]. Because of that it is the advantage also is that you can actually make progress with the information you get there [during the MI-training], you can apply it” (Pod08) |
| - 1. **Multimodal training method** | “There was also room for interaction, so if there were any questions you could just raise your hand and fill in. So you didn’t have to listen for 1.5 or 2.0 hours continuously…so the interaction was good. We had to do exercises ourselves, I also thought that was good. It was just a good mix of theory and practice” (Pod14)  “…I also liked that we didn’t had a too large group, so that everyone actually participated very actively during the meetings” (Pod07)  "During the course, he [the trainer] really listened to what we were already doing and what we might already be able to do. Then he just adapted it [the MI-training], he could adjust it [the MI-training] à la minute. I thought that was very nice” (Pod12) |
| - 1. **Importance of repeating MI-training information** | "I received then an email as refresher of that course [the MI-training]. I save the content of that email, and every now and then I think what did it mean again. Sometimes it fades again, and then I find it useful to refresh it [the MI-training content]” (Pod17)  "I’ve to say that I also liked the booster training via teams. That is also the power of repetition, and you notice that with a lot of things, you just got a little better in it [the application of MI]” (Pod04) |
| - 1. **Points of improvement** | |
| - - 1. Applicability of MI | "Sometimes I thought it [the MI-training] was kept so broad, that I found it very difficult to project it [the MI-training content] on my own profession. I just found that difficult, if it had been a little more specific to our target group, then I would probably have experienced it differently…That’s because sometimes I really felt that is wasn’t always applicable or something” (Pod02)  “I missed the feedback on that what you applied in practice” (Pod13) |
| - - 1. Multimodal training method | “Well, of course we had him [the MI-training] through Teams, which I thought was very well arranged…However I personally like face-to-face courses better, because it just makes it a bit easier to spar” (Pod16)  “I have to stay that it was a lot of new material. In the beginning I really thought…gosh what a lot of information…Sometimes the days were long but that’s part of things like that [training/courses]. However, I am more a practical person than a congress person, so such a day is long” (Pod05) |
| - - 1. Importance of repeating MI-training information | “If you want to implement motivational interviewing well, than you should actually have a booster session now and then over a longer period of time” (Pod05)  “I thought there was a bit of repetition in it [the MI-training]” (Pod12) |
| 1. **Podiatrist’s experiences with MI in practice** | |
| - 1. **Partnership** | “Well the collaboration…I had the idea for myself that I actually always worked together , to a certain extent, and perhaps also a bit more unconsciously than consciously” (Pod15)  “That motivational interviewing is aimed that you have an equal conversation” (Pod10)  “I find it difficult to get the repair reflex out” (Pod03)  "The combination of working together, and providing applicable solutions , or thinking along, is often very experienced as very pleasant by them [the patients]” (Pod09)  “The combination of working together and providing an applicable solution, or thinking along, they [the patients] experience that often as very pleasant and it also works much easier…” (Pod10)  “So I think that if people start to change themselves, already want something, then it is of course much easier to go with that…but a patient with diabetes who actually does not know what he wants and has no idea what is good for him, that is a different story” (Pod10)  “Give confirmation that it is going very well, and try to ensure that he [the patient] also remains positive and motivated about that, so that he is also in the long term motivated” (Pod13)  “Well I recognise that…you just send it [information]…but without reaching that person” (Pod17)  “So I often try to show those people with an introductory meeting at the pedorthist what the possibilities are [with regard to orthopaedic shoes], to plant a seed there” (Pod05)  “It’s just a different approach…you always run into the same thing, you give people advice, but they don’t do anything with it” (Pod11) |
| - 1. **Change talk** | “You give them [the patients] insight in what is actually good for them, so they think about it themselves, instead of you saying that you [the patient] should wear those shoes. That you say: ‘So you prefer you current shoes and getting a wound, instead of keep using the good shoes and stay ahead of a wound, so you don’t have to go to the hospital?’ Then you see that they [the patients] start thinking and they say: ‘I hadn’t thought about it like that…By giving them the facts, you make them think and realise that they didn’t looked at it that way” (Pod03)  "It’s nice, you can go a bit more in-depth with such conversations. It makes it possible that you sometimes can look at certain topics in a better way by asking question about why and how. In the end that has really been an enrichment for me” (Pod10)  "I noticed that evocation, it is very interesting and I also think it is very meaningful... but that has not always been my way to do it…I noticed that I have trouble applying that” (Pod04)  “It is of course a target group that very often does not know how and what, and then you have to elicit that” (Pod02) |
| - 1. **Acceptance** | “…I also think that acceptance is very logical…” (Pod15)  “…Accepting if a patient does not want to cooperate, I still have a hard time with that” (Pod05)  “And if it really doesn’t work right away, then I’ll just take a little longer and let the patient come back sometime or give them more time to think about it…The more compelling I come across, or them more I demand of the patient, the greater the patient’s shield becomes against me, so therefore I give people a little more rest and time [to think]” (Pod16) |
| - 1. **Compassion** | “Well I think that is also one of the reasons why you went working into care I think. To help someone actually, you don’t do that to become better of it yourself” (Pod15) |
| - 1. **Ask open questions** | “Especially asking questions, asking open questions is more difficult than I thought, because you actually think you always ask open questions, but you actually ask much more closed questions [than you think]. And if you have someone who is very closed off and you ask closed questions, you actually get very little information [from/about the patient]” (Pod03)  "But if you really have to take the last step and ask him what would you do, what’s so bad about changing something? I found that last step the most difficult [one] to take, and also to get the patient on board” (Pod12)  “I think I am very handy with certain questions that we have been given” (Pod06)  “So I found that difficult, because at some point you feel a bit of a whiner when you come up with those extra questions every time” (Pod12) |
| - 1. **Applicability of MI** | “I actually never had any trouble motivating someone to wear orthopaedic shoes. That was never a problem for me. For some colleagues it was a life-changing thing, but not for me, I had no problem with it” (Pod01)  “I actually already did it a bit, in the same way, but without a name” (Pod09)  “If I have MI-conversation, then I try to use the tips and tricks [from the MI-training], although I it feels very unnatural…because you’re really going to question things, and then I just switch back to the normal approach” (Pod01)  “I think you can use it [MI[ then [in combination with a foot examination] much more into your story. That’s why feels more natural” (Pod02)  “I still use it [MI] a lot, also besides the study” (Pod16)  “Well sometimes it is difficult [to apply MI], it also depends a little bit on who is sitting opposite of you…With one patient I succeed very well in pointing out to them that their decision is wrong without coming into conflict, but with other patients you just keep that it there is always friction” (Pod14)  “I found it easier when it was a patient I already knew in practice” (Pod02)  “Every now and then you got a patient that you didn’t know, and sometimes I found it difficult to start applying MI right away, because you actually have no idea who or what you are dealing with” (Pod02)  “I think especially with the slight more closed patient. It costs a lot of energy to get out that part that you actually want to hear [from him/her]” (Pod07)  “It’s not that I am afraid, but I am especially curious or find it exciting how someone reacts…especially a certain age category. I am more afraid of [patients who are] 50+ than people who are younger” (Pod06)  “With certain difficult patient where communication does not run completely smoothly, then you would rather think of applying MI. You think about, how can I collaborate with the patient, so that we can work together towards one goal” (Pod13)  “I think it’s difficult to be very conscious using it [MI] all the time…that you very easily do what you were used to do…because you are purely focused on the complaint…then you will do those things that you have to do actually” (Pod05)  “For example, there is a home situation in which people very quickly say ‘I’ll take my shoes off’. I find it very difficult to motivate those people, because I understand why those people take their shoes off” (Pod04)  “Then you think maybe I shouldn’t do it [apply MI] next time, that’s the risk” (Pod12)  “I have gained new insights, but then you might also come to the applicability…I find that difficult…I think there are sometimes other conversation techniques that are better” (Pod15) |
| - 1. **Behavioural change for podiatrist** | “I notice that in myself, I am now very aware of that, that I very quickly tend to offer solutions immediately, while you very often miss the point” (Pod02)  “I’ve been working as a podiatrist for 10 years so you’re also completely in your own ways and your own things…it is indeed a complete change, the application [of MI] itself is still quite difficult” (Pod15)  “In the beginning it was a bit of a struggle about what exactly I am going to say, which sentences I am going to use, but if you do that more often, you quickly become handy” (Pod14)  “I noticed that if you’re not working with it [applying MI] every day, it quickly disappears into the background” (Pod03)  “I do try to [apply MI] more and more…I don’t always think about it yet, but the more you focus on it, the more you become aware of it” (Pod13)  “I am now a little more aware of motivational interviewing. I work with it a little more consciously and I am more aware that it exists” (Pod17)  “But I do apply it [MI] continuously, but am not so strongly aware that I do so. I do not think in the morning, now I am going to apply MI, it has become my own” (Pod08)  “I have absolutely not been involved with the patient exactly with those four basic principles” (Pod08)  “I did pick my things up, I won’t say I made a 100% copy of every I learned. But I just took out the details that I thought I can apply myself and I can work with” (Pod01) |
| - 1. **Added value of MI** | |
| - - 1. Change talk | “You also have to be able to argue it [wearing orthopaedic shoes] very well, I think, and be able to substantiate it. So I do think it is an added value, but then especially for the people who have difficulty with that” (Pod01)  “It is of added value for a lot of patients who are still just a bit in doubt…and thus show themselves that they can change themselves instead of that I [the podiatrist] am always the one for them to say to do this and adjust to that, and then it’ll be fine” (Pod10)  “That the patient chooses: I choose this, or don’t choose it” (Pod08)  “The behavioural changes is actually seen in the patient who, through the last [MI-] conversation I had with him, was actually suddenly willing to make an appointment with an pedorthist, which he never wanted in the past…” (Pod14) |
| - - 1. Patient dependent | “Because this is a group that is sometimes difficult to motive, and that was also our first feeling, how should we motivate those people. But I think if that’s the feeling you have about something, that is also the best target group to motivated in this way. I do think it is the right target group [to apply MI on]” (Pod04)  “I think that the patients who are open to it, that they really see the benefit of it” (Pod07)  “He’s motivated, I know him too, it’s not that you haven’t seen him enough, you know where you started, then I really think this is so useless” (Pod05)  “No, I’ve no idea whether the shoes fit well, whether they are worn…on the one hand you also shouldn’t have patients that you know too well or that you know they are adherent, but when only seeing a patient for the very first time, and never seeing that patient again after such a conversation, I question the added value” (Pod05) |
| - 1. **Dealing with resistance to orthopaedic shoes** | “You also accept that they feel that resistance. At least, you indicate that you think it is logical that they experience it that way” (Pod15)  “You first explain what is going on, what the problem and diagnosis is, what a solution could be, and what concessions they have to make in functionality, and how the shoes look like. And I always explain that scale in function and how it looks like…if you make it negotiable you don’t actually get a problem or a weird reaction from them” (Pod08) |
| 1. **Patient’s experiences observed and mentioned by the podiatrist** | |
| - 1. **Partnership** | “…People always like to be flexible themselves and that you [as podiatrist] think on the wish of the patient, that that wish is heard and you [the podiatrist] not just sit there as a listener. That is what all people like I think” (Pod17)  “I think it takes time of getting used to [the application of MI], especially for patients…because they come to you to solve their complaint, or arch support or whatever. And then you suddenly turn it around, by letting them do things themselves, and…they probably didn’t expect that either. Some do, some don’t” (Pod09)  “Especially because he received the confirmation from me that he was doing well…I got the feeling that he liked to get the confirmation…” (Pod13)  “That you notice that as the conversation progresses, their attitude becomes more open, that their hands are no longer crossed and that they have a different facial expression” (Pod16)  “I notice that especially the new way of reporting, we now have a different way of reporting for diabetes patients, ensures that they really have the idea of what a lot of questions. We now really have to tackle many things step by step, so ask a lot of questions about how things are going with the pedicure, but also other questions. That it is a bit too much for some [patients], and then they have the feeling is that all necessary?” (Pod10) |
| - 1. **Change talk** | “Because I had patients who said at the end [of the conversation] said for example: ‘okay…so I can do something about it myself’” (Pod12)  “Two or three weeks ago I had a conversation with someone, and he came in and he said that he was so glad that I started such a conversation with him, that it really made him think. And that was actually going a lot better, even noticed that his walking was getting better” (Pod07)  “Sometimes I’ve the impression that the moment you start talking about the shoes by patient with diabetes, they find it whining or an obligation. Then you may also elicit something that it feels confrontational [for the patient] and that that is experienced as negative” (Pod15) |
| - 1. **Other aspects** | “But I think it took some time getting used to it [the application of MI] for the patient, because they just come [to us] with an expectation [about the appointment] and when we don’t meet that expectation, it will be different for them” (Pod09)  “I also had a patient with diabetes, who really thought it was all nonsense that I asked those question. Therefore the conversation was really uncomfortable” (Pod12) |
| 1. **Recommendations** | |
| - 1. **Application MI by all podiatrists** | |
| - - 1. Partnership | “…A patient comes to you [the podiatrist] with something [a problem], and wants to go somewhere [a solution], then you [the podiatrist] are there to guide that patient there. I don’t think it will work if I just say: ‘you have to do this and that’, patients already have to do so much. I think it works better if you [the podiatrist] just listen very carefully to what the patient needs, and that you work with the patient to find a solution, and not because you are convinced that there is only one way that works” (Pod11)  “…when applying it [MI] in practice that simply allows patients to get very different insights, which actually increases patients’ adherence…” (Pod13)  “It [the application of MI] improves insights from the patients themselves. It’s not that I’m as podiatrists going to fix it for you, but I’m the podiatrists and I can help you, so that we can do together the best for you…” (Pod04) |
| - - 1. Change talk | “…eliciting those questions from people themselves, so that you let them think for themselves. And I have the idea that by thinking they also give a different answer than they would in the first place [without thinking about it themselves first]…” (Pod03) |
| - - 1. Other aspects | “It is indeed because I think that you also become very aware of the way you conduct a conversation in the first place. You really don’t’ know that [what you are doing], you just do what you think is right. Because of the training I really found out in what way I conduct conversations, and in what way I could improve them” (Pod12)  “I think that it [the MI-training] is an added value for everyone to have a bit of background knowledge [about conversation techniques]…” (Pod05)  “It is a very valuable addition about how you deal with patients…to be able to have better conversations with a patient…” (Pod17)  “… it [the application of MI] also gives you insights about a piece of self-knowledge, but also about the knowledge of others. So you can quickly see from others whether they apply only sustain talk or also want to change…” (Pod10)  “If I at least think for myself, I also have several [conversation] techniques that I find interesting. Now [in this training] it was really just motivational interviewing, but I believe…that there are more options. So I personally think that not one option is the right one, sing you also have to deal with many different types of people” (Pod10) |
| - 1. **Include MI in the primary podiatry education** | “When I think back to the beginning of my career, I recognise that I often had problems with patients…I feel like I had those kinds of situations more in the beginning than in recent years. And I think that has a lot to do with the fact that I do indeed think more along with the wishes of the patient…So get the expectations [from your patient] very clear” (Pod17)  “If I look at the podiatry training, it should have been included in three…then you can make it come back more often” (Pod03)  “I do think that, imagine if it [the MI-training] was really a one-year course, that would be even more effective” (Pod10)  “I don’t know if it [MI] should be in the podiatry training…maybe you should do it one or two years afterwards, so that you have rest in the treatment room, that you know what to do, and that you also have time to apply motivational interviewing...It is the most desirable situation and you should definitely to it, but I don’t know whether that will work, or whether that it is feasible. They [the students] are happy if they can ask a question about the correct diagnosis, and I wonder when I see the fourth-year students start working, I don’t think it is feasible to apply motivational interviewing complete correctly, but of course they can already get tips” (Pod08)  “I especially think about the podiatrist who are already familiar in practice, because when you are just starting and you are still looking how does everything work…they actually thought that it was a lot [already they had to think about]…after six months of working, than I think it is actually a very good moment to get that course, because then you are just a bit in your rhythm…then you can train your conversation techniques very nicely” (Pod07) |
